# Mitigating preventable chronic disease: Progress report of the Cleveland Clinic's Lifestyle 180 program

**DOI:** 10.1186/1743-7075-8-83

**Published:** 2011-11-23

**Authors:** Elizabeth HW Ricanati, Mladen Golubić, Dongsheng Yang, Leif Saager, Edward J Mascha, Michael F Roizen

**Affiliations:** 1 Wellness Institute, Cleveland Clinic, 1950 Richmond Road, TR2-341, Lyndhurst, OH 44124, USA; 2 Quantitative Health Sciences, Cleveland Clinic, 9500 Euclid Avenue, Cleveland, OH 44195, USA; 3 Outcomes Research, Cleveland Clinic, 9500 Euclid Avenue, Cleveland, OH 44195, USA

**Keywords:** Lifestyle, Nutrition, Exercise, Stress Management, Chronic disease, Obesity, Hyperlipidemia, Hypertension, Diabetes

## Abstract

**Background:**

Poor lifestyle choices are key in development and progression of preventable chronic diseases. The purpose of the study was to design and test a program to mitigate the physical and fiscal consequences of chronic diseases.

**Methods:**

Here we report the outcomes for 429 participants with one or more chronic conditions, including obesity, hypertension, hyperlipidemia and diabetes mellitus, many of whom had failed traditional disease management programs, who enrolled into a comprehensive lifestyle intervention. The Lifestyle 180 program integrates nutrition, physical activity and stress management interventions and was conducted at the Wellness Institute of the Cleveland Clinic, United States. An intensive 6 week immersion course, with 8 hours of group instruction per week, was followed by 3 follow-up, 4 hour-long sessions over the course of 6 months.

**Results:**

Changes in biometric (weight, height, waist circumference, resting heart rate and blood pressure) and laboratory variables (fasting lipid panel, blood glucose, insulin, hemoglobin A1c, ultra sensitive C-reactive protein) at 6 months were compared with baseline (pre-post analysis). At week 30, biometric and laboratory data were available for 244 (57%) and 299 (70%) participants, respectively. These had a mean ± SD reduction in weight (6.8 ± 6.9 kg, P < 0.001), waist circumference (6.1 ± 7.3 cm, P < 0.001), glucose (4.5 ± 29.6 mg/dL or 0.25 ± 1.64 mmol/L, P = 0.009), triglycerides (26.4 ± 58.5 mg/dL or 0.30 ± 0.66 mmol/L, P < 0.001), low-density lipoprotein cholesterol (LDL) (7.9 ± 25.1 mg/dL or 0.2 ± 0.65 mmol/L, P < 0.001), hemoglobin A1c (HgbA1c) (0.20 ± 0.64%, P = 0.001), insulin (3.8 ± 11 microU/ml or 26.6 ± 76.4 ρmol, P < 0.001) and ultra sensitive C-reactive protein (US - CRP) (0.9 ± 4.8 mg/dL or 7.3 ± 40.2 nmol/L, P = 0.012), an increase in mean high-density lipoprotein cholesterol (HDL) (3.7 ± 8.4 mg/dL or 0.1 ± 0.22, P < 0.001), and decreased use of medications.

**Conclusion:**

Implementation of a comprehensive lifestyle modification program among adults with common chronic conditions results in significant and clinically meaningful improvements in biometric and laboratory outcomes after 6 months.

## Background

Interactions between lifestyle and genetic factors cause the development and progression of a spectrum of chronic conditions, including obesity, type 2 diabetes mellitus, hypertension, cardiovascular disease and several types of cancer. For some of these major causes of mortality, not only in Western societies but globally, more than 80% of attributable risks may be related to environmental, primarily lifestyle factors [1,2]. In a recent prospective study, participants who were successful in maintaining body mass index below 30, not-smoking, exercising about 3.5 hrs per week and eating mostly plant-based diets with limited amount of meat had a 78% lower risk of developing chronic disease than those without a healthy lifestyle factor [3]. It is, therefore, not surprising that health-promoting lifestyle behaviors are prominently included in practice guidelines for prevention or management of most chronic conditions [4]. Several groups of investigators have successfully used comprehensive lifestyle interventions for treatment of chronic conditions, including coronary artery disease, metabolic syndrome and type 2 diabetes mellitus [5-9].

Without addressing the key underlying causes of modern chronic conditions, that is, lifestyle factors, it is hard to imagine that optimal healthcare can be delivered for all citizens at reduced cost and in a long-term, sustainable fashion [10]. At the Cleveland Clinic, in late 2008 Lifestyle 180^® ^was launched with the goal of mitigating the physical and fiscal consequences of preventable chronic diseases. This program employs a system of class-based instruction and ongoing follow-up for participants with common chronic diseases around three key areas: nutrition, physical activity, and stress management. Here, we report findings for participants who completed six months of the program.

## Methods

### Participants

Participants who had at least one of 8 chronic conditions (obesity, hypertension, hyperlipidemia, diabetes, non-alcoholic fatty liver disease, multiple sclerosis, early stage breast or prostate cancer) and had been seen by the primary care physician within three months of enrollment and had provided a note of medical clearance were eligible for participation in the program. Patients who smoked, first enrolled in the Cleveland Clinic's tobacco cessation program before being permitted to enroll in Lifestyle 180. Patient recruitment was threefold: self-referral, physician-referral or sponsorship by local self-insured employers. Prior to starting Lifestyle180, participants completed an intake packet that included past medical and surgical histories, pertinent social history, medication use, psychosocial questionnaires and a 1-day food diary.

Considering guidelines from the Third Report of the National Cholesterol Education Program (NCEP) Expert Panel on Detection, Evaluation, and Treatment of High Blood Cholesterol in Adults (Adult Treatment Panel III) [11], the diagnosis of hyperlipidemia was used for participants with:Triglyceride ≥ 150 mg/dL; Total cholesterol ≥ 200 mg/dL; LDL cholesterol ≥ 130 mg/dL; HDL cholesterol < 40 mg/dL for males and < 50 mg/dL for females, or normal lipids but participant was taking anti-hyperlipidemia medication. The diagnosis of hypertension was used for participants with blood pressure ≥ 140/90 mmHg at each of two or more visits taken on separate days or for participants with a normal blood pressure but taking antihypertensive medication. For obesity diagnosis, we used the recommended classifications for BMI adopted by the National Institute of Health and World Health Organization [12]. Overweight -- BMI ≥25.0 to 29.9 kg/m_2_; Obesity -- BMI ≥30 kg/m_2_; Obesity Class I -- BMI of 30.0 to 34.9 kg/m_2_; Obesity Class II -- BMI of 35.0 to 39.9 kg/m_2_ and Obesity Class III -- BMI ≥40 kg/m_2_. Diagnostic criteria for pre-diabetes and diabetes were from the American Diabetes Association, Diagnosis and Classification of Diabetes Mellitus from January 2011 [13]. Pre-diabetes was indicated by fasting plasma glucose of 100 mg/dL to 125 mg/dL or hemoglobin A1c 5.7-6.4%. For diabetes diagnosis we used fasting plasma glucose of ≥126 mg/dL or hemoglobin A1c ≥ 6.5%. The International Diabetes Federation criteria were used for diagnosis of metabolic syndrome [14]. A person to be defined as having the metabolic syndrome must have central obesity (waist circumference ≥ 94 cm for men and ≥ 80 cm for women) plus any two of the following four factors: raised triglyceride level ≥ 150 mg/dL, reduced HDL cholesterol (< 40 mg/dL in males and < 50 mg/dL in females, raised blood pressure (systolic BP ≥ 130 mm Hg or diastolic ≥ 85 mm Hg or raised fasting plasma glucose ≥ 100 mg/dL.

Lifestyle intervention was additive to the treatment plan of all participants who continued medical care from their pre-existing primary care physicians. Participants were instructed to contact their primary physician to adjust medications when deemed necessary, for example when participants' blood pressure and glucose readings started to decrease into subnormal ranges. All hypertensive or diabetic patients were encouraged to monitor daily their blood pressure or fasting glucose, respectively.

An initial 48 hrs of lessons curriculum was taught twice per week, in four-hour sessions each over the first six weeks, called an Immersion Phase. During this period, the first four-hour session of the week was divided into 60 minutes of participant physical activity and 90 minutes of participant cooking and nutrition as well as stress management classes, while during the second session of the week, 60 minutes period was devoted to participant exercise and practice of cooking/nutrition, stress management and biometric one-on-one sessions each. The follow-up phase included three components to help maintain behavioral changes. These included 1) on-site classes at weeks 10, 18 and 30 that followed the same four-hour structure as occurred during the Immersion phase on the second day of the week, 2) a weekly e-mail newsletter and 3) the buddy system. Buddies were assigned within each group during the initial six-week session and were asked to keep in daily contact.

### Intervention

Lifestyle 180 was designed by taking into account findings that behaviorally-based lifestyle modification approaches increase the likelihood of sustained changes [15].

Multiple modalities are incorporated into the curriculum, including face-to-face interactions, individually-tailored and group-tailored education sessions and counseling to encourage internal motivation. Participants receive a short syllabus written at 5th grade level. Three key, closely interconnected, elements of the Lifestyle 180 curriculum included nutrition, physical activity and stress management. Participants enrolled since October 2008 until April 2010 were included in this analysis.

### The Nutrition Module

The primary goal for the nutrition curricular component was to alter patients' diets to foster measurable improvements in biometric and laboratory disease markers. Nutritional aspects of the program most closely align with the Mediterranean diet [16]. The Lifestyle 180 program eliminates trans fats as well as added sugars and syrups, limits saturated fat to < 4 gm/meal (fish, skinless chicken and skinless turkey breast being the only types of animal food sources) and substitutes only 100% whole grains/grain products for processed grain foods. Besides complex carbohydrates, Lifestyle 180 nutritional approach promotes an increase in intake of plant foods to provide a spectrum of phytochemicals exerting diverse beneficial biological functions [17]. Improved glycemic control may help reverse or stabilize consequences of diseases such as diabetes and hypertension and possibly beneficially affect aging by activation of the human transcriptional machinery homologue of Caenorhabditis elegans DAF-16 gene complex [18-20]. This food strategy is also likely to promote weight loss, although this is not the primary goal of Lifestyle 180 [21].

The nutritional component of Lifestyle 180 was team-taught, with a chef and a registered dietitian. First, the chef worked with participants in the teaching kitchen. In an adjoining dining room/classroom, while participants were eating a meal they helped prepare, the dietitian conducted a seminar and guided a discussion about the nutritional aspects of what they had cooked as well as a covered topic of the day. To motivate participants to change how and what they eat, they were engaged into meal preparation in the kitchen. Gaining confidence with the kitchen equipment and different types of foods as well as learning how to manage time during meal preparation were considered important elements of the efforts to increase the likelihood that participants will change their dietary habits. As part of the nutrition education of participants, one class during the Immersion Phase involved a grocery store experience. In this environment, participants learned how to read nutrition and ingredient labels, what to avoid in packaged foods and how to "shop" a grocery store (i.e. what aisles to avoid, which to frequent).

### The Physical Activity Module

The goal of the physical activity component of Lifestyle 180 was to increase both endurance and strength of participants. Current guidelines for adults recommend at least 150 minutes of physical activity per week to maintain fitness levels and at least 200 minutes per week for waist circumference reduction and weight loss [22]. An exercise instructor led the class in the Lifestyle 180 Fitness Center that included progressive cardiovascular and endurance training as well as resistance training. Patients chose between treadmills, elliptical machines, stationary bicycles or Concept Rowers. Resistance equipment includes dumbbells (2-15 lb each), stretch bands (resistance tubing: extra light, light, medium and heavy) and medicine balls (2-15 lbs each). The goal was to teach participants how to engage in physical activities in a safe and effective manner and to enable them to gain the skills to exercise and progress on their own, including resistance exercise. They were taught that increased resistance type activity helps lose and maintain weight loss. In particular, visceral adipose tissue is preferentially lost, while maintaining muscle mass [23,24]. Patients were encouraged to wear pedometers that were provided at start of the Program, with a goal of 10,000 steps per day and to record their steps on a daily diary sheet [25,26]. These data were used to monitor participants' progress in exercise.

### The Stress Management Module

This component was team-taught, with a restorative yoga therapist and a behavioral health specialist. To sustain healthy lifestyle modifications, behavioral interventions were implemented during the course of the program. Those included goal setting, keeping records of lifestyle practices, social support, cognitive restructuring and problem solving. Patients learned meditation and mindfulness practices and performed simple yoga poses initially sitting or standing and at later stages of the program lying down. Regular elicitation of the "relaxation response", the physiological opposite of the "fight or flight response", may reduce psychological distress and improve medical symptoms in patients with any of a wide array of medical conditions [27]. These practices were integrated into the program to teach participants how to be present in the moment, more aware of their thoughts and feelings to recognize old thoughts and patterns that no longer serve them and release those unhelpful ways of thinking and believing. The focus was on accountability and compliance with the program and for that purpose a daily diary checklist was reviewed in each class by the behavioral health specialist and weekly by the physician and nurse case manager during one-on-one sessions with each participant.

### Outcome Assessment

To measure the effectiveness of the Program, we established an IRB-approved patient registry (Cleveland Clinic Foundation IRB number 09-154) to collect biometric, metabolic and psychosocial data. At each one-on-one session, body weight, waist circumference, resting heart rate and blood pressure were recorded while height was measured only at the start of the program for body mass index (BMI, calculated as weight in kilograms divided by height in meters squared) calculation. Participants were in socks and lightly dressed when body weight and height were measured. Waist circumference was measured by a tape measure in the midspace between the lowest costal margin and the iliac crest. Blood pressure was measured by the standard manual protocol using sphygmomanometer. In addition, patients were queried weekly regarding their medications and changes in medications were recorded. Patients and their physicians were given the results of the biometrics and metabolic variables as they were obtained. At the beginning of the program, participants completed a 1-day food diary that was analyzed by a registered dietitian and reviewed with the participant during week one of the Immersion phase.

Laboratory measures included total cholesterol (to convert from mg/dL to millimoles per liter, divided by 38.67), triglycerides (to convert from mg/dL to millimoles per liter, divided by 88.57), low-density lipoprotein cholesterol (LDL-C), high-density lipoprotein cholesterol (HDL-C) by standard enzymatic methodology, hemoglobin A1c (HgbA1c) (in percentages %) by turbidimetric inhibition immunoassay, insulin (to convert from microIU/ml to picomoles per liter divided by 6.94) by chemiluminescence immunoassay, ultra sensitive C-reactive protein (US-CRP) (to convert from mg/L to nanomoles per liter divide by 8.45) by immunoturbidometric assay and fasting plasma glucose (to convert from mg/dL to millimoles per liter divided by 18) by glucose hexokinase method. These values were measured at the beginning of the study (week 1) and at weeks 6 and 30. Hemoglobin A1c was not evaluated at week 6.

### Statistical Methods

Continuous variables were presented as mean ± SD and categorical variables as n (%). For univariable analyses, changes in outcomes at 6 and 30 weeks from baseline were assessed with the paired t-test. Analysis of covariance was used to assess the relationship between weight loss and baseline variables obesity status, diabetic status, hyperlipidemia, hypertension, metabolic syndrome while adjusting for age, gender and payment options. We also report the percentage of participants who experience weight loss of 2.3 kg or more, that is, beyond the change expected with normal daily weight fluctuation [28]. Results estimate changes from baseline and not efficacy of the wellness program. Patients with incomplete data at either baseline or follow-up were excluded when assessing change or percent change from baseline, as were patients with missing covariables for multivariable models. No imputation of data of missing data values was done. All reported P values are two-sided and the significance level was 0.05 for each hypothesis; a Bonferroni adjustment for multiple comparisons over time within each hypothesis was made (i.e., significance criterion at each of 6 and 30 weeks was 0.05/2 = 0.025). SAS statistical software, Carey, NC, was used for all analyses.

## Results

### Characteristics of participants

Baseline characteristics of participants are shown in Table 1. The mean age of 429 participants was 52 years, more participants were female (65%), 32% of participants had diabetes, 59% had hyperlipidemia, 63% had hypertension, 80% were obese and 59% fulfilled the diagnostic criteria for metabolic syndrome and 40% for pre-diabetes [13,14]. The number of participants with other chronic conditions, including breast and prostate cancer, fatty liver and multiple sclerosis was much smaller (less than 5%).

**Table 1:** Basic Characteristics of Patients (N = 429)^a^

Variable	Statistics^b^
Female N (%)	278 (65)
Obese (BMI > 30) N (%)	345 (80)
Pre-diabetes N (%)	170 (40)
Diabetes N (%)	139 (32)
Hyperlipidemia N (%)	255 (59)
Hypertension N (%)	270 (63)
Metabolic syndrome N (%)^c^	247 (59)
Age (years)	52 ± 11
Height (cm)	168 ± 9
Weight (kg)	103 ± 25
BMI (kg/m^2^)	37 ± 8
Waist (cm)^d^	114 ± 18
SBP (mmHg)	132 ± 15
DBP (mmHg)	82 ± 10
Heart rate (bpm)	78 ± 11
Fasting Glucose (mg/dL)^e^	108 ± 35 (6.0 ± 1.9 mmol/L)
Triglycerides (mg/dL)^f^	137 ± 81(1.5 ± 0.9 mmol/L)
Cholesterol (mg/dL)^f^	187 ± 39 (4.8 ± 1.0 mmol/L)
HDL (mg/dL)^f^	51 ± 15 (1.3 ± 0.4 mmol/L)
LDL (mg/dL)^e^	109 ± 33 (2.8 ± 0.9 mmol/L)
HgbA1c (%)^j^	6 ± 1
Insulin (microU/mL)	19 ± 18 (131 ± 122 ρmol)
US-CRP (mg/dL)	5 ± 8 (45 ± 66 nmol/L)

Abbreviations: BMI, body mass index; SBP, systolic blood pressure; DBP, diastolic blood pressure; HDL, high-density lipoprotein cholesterol; LDL, low-density lipoprotein cholesterol; HgbA1c, hemoglobin A1c; US-CRP, ultra sensitive c-reactive protein.

^a ^Insulin (N = 271) and US-CRP (N = 285) only measured beginning June 2009.

^b ^N (%) or mean ± SD.

^c, d, e, f, j ^indicate 7, 1, 9, 6 and 34 missing data points, respectively.

At week 6 (the end of the Immersion Phase) 404/429 (94%) of participants had biometric measurements taken and 396/429 (92%) had labs drawn, while at week 30, 244/429 (57%) returned on time for a follow up and 299/429 (70%) had labs drawn.

There were few, but important differences in mean baseline variables between these participants who had either biometric or metabolic data available at their week 30 visit and those who did not, six week variables, or change from baseline to six weeks. Specifically, those who had week 30 biometrics completed were less likely to have obesity and greater lowering of mean cholesterol and LDL at six weeks than those who did not have week 30 data. Those who had week 30 cholesterol data (as an indicator of blood work being completed) were more likely to have greater lowering in mean weight, triglycerides, cholesterol and LDL than those with cholesterol levels not available (Table 2).

**Table 2:** Association between compliance at 30 weeks and baseline conditions^a^

Variable	30-week Weight available		*P* value	30-week Cholesterol available		*P *value
	
	Yes (N = 243)	No N = (186)		Yes (N = 295)	No (N = 128)	
**Baseline conditions:**						

Diabetes N (%)	176 (72)	133 (72)	0.97	220 (75)	88 (69)	0.26
Obesity (BMI> 30) N (%) N (%)	185 (76)	160 (95)	0.011^b^	232 (79)	108 (84)	0.17
Hyperlipidemia N (%)	151 (62)	104 (56)	0.19	185 (63)	68 (53)	0.065
Hypertension N (%)	155 (64)	115 (62)	0.68	193 (65)	73 (57)	0.10
Metabolic syndrome N (%)	135 (56)	112 (61)	0.33	175 (60)	71 (56)	0.47

**Biometrics at week 6:**						

Weight (kg)	95 ± 22	105 ± 26	< 0.001^b^	96 ± 22	105 ± 26	0.002^b^
Waist (cm)	107 ± 15	112 ± 17	0.002^b^	108 ± 15	112 ± 18	0.046^b^
Body mass index (kg/m^2^)	34 ± 7	37 ± 8	< 0.001^b^	34 ± 7	37 ± 8	0.004^b^
Systolic blood pressure (mmHg)	126 ± 13	127 ± 15	0.66	126 ± 13	127 ± 15	0.69
Diastolic blood pressure (mmHg)	79 ± 8	79 ± 9	0.78	79 ± 8	79 ± 9	0.90
Heart rate (bpm)	71 ± 11	73 ± 12	0.056	71 ± 11	74 ± 12	0.014^b^

**Metabolic variables at week 6:**						

Glucose (mg/dL)	101 ± 25	104 ± 27	0.32	101 ± 24	104 ± 30	0.40
(mmol/L)	5.6 ± 1.4	5.8 ± 1.5		5.6 ± 1.3	5.8 ± 1.7	

Triglycerides (mg/dL)	102 ± 49	115 ± 54	0.02^b^	103 ± 49	118 ± 57	0.023^b^
(mmol/L)	1.2 ± 0.6	1.3 ± 0.6		1.2 ± 0.6	1.3 ± 0.6	

Cholesterol (mg/dL)	164 ± 35	170 ± 38	0.11	164 ± 36	171 ± 37	0.08
(mmol/L)	4.2 ± 0.9	4.4 ± 1.0		4.2 ± 0.9	4.4 ± 1.0	

High-density lipoprotein (mg/dL)	51 ± 15	48 ± 13	0.08	50 ± 14	49 ± 15	0.33
Cholesterol (mmol/L)	1.3 ± 0.4	1.2 ± 0.3		1.3 ± 0.4	1.3 ± 0.4	

Low-density lipoprotein (mg/dL)	92 ± 29	99 ± 34	0.06	93 ± 30	99 ± 33	0.11
Cholesterol (mmol/L)	2.4 ± 0.7	2.6 ± 0.9		2.4 ± 0.8	2.6 ± 0.9	

**Change in biometrics from baseline to six weeks:**						

Weight (kg)	-4.0 ± 2.5	-3.6 ± 2.8	0.16	-4.1 ± 2.6	-3.1 ± 2.7	< 0.001^b^
Waist (cm)	-4.0 ± 4.1	-4.4 ± 4.2	0.36	-4.2 ± 4.1	-4.0 ± 4.4	0.66
Body mass index (kg/m^2^)	-1.4 ± 0.9	-1.3 ± 1.0	0.12	-1.5 ± 0.9	-1.1 ± 0.9	< 0.001^b^
Systolic blood pressure (mmHg)	-5.1 ± 15.7	-5.1 ± 15.2	0.98	-5.8 ± 15.1	-3.7 ± 16.6	0.24
Diastolic blood pressure (mmHg)	-2.7 ± 8.6	-3.9 ± 10.9	0.24	-3.1 ± 9.2	-3.5 ± 10.7	0.75
Heart rate (bpm)	-4.7 ± 1.7	-6.2 ± 11.2	0.20	-5.4 ± 10.9	-5.3 ± 12.8	0.92

**Change in metabolic variables from baseline to six weeks:**						

Glucose (mg/dL)	-6 ± 24	-6 ± 25	0.96	-6 ± 22	-7 ± 29	0.67
(mmol/L)	-0.3 ± 1.3	-0.3 ± 1.4		-0.3 ± 1.2	-0.4 ± 1.6	

Triglycerides (mg/dL)	-31 ± 55	-23 ± 51	0.12	-32 ± 55	-17 ± 49	0.010^b^
(mmol/L)	-0.4 ± 0.6	-0.3 ± 0.6		-0.4 ± 0.6	-0.2 ± 0.6	

Cholesterol (mg/dL)	-25 ± 26	-17 ± 25	< 0.001^b^	-24 ± 28	-14 ± 19	< 0.001^b^
(mmol/L)	-0.7 ± 0.7	-0.4 ± 0.6		-0.6 ± 0.7	-0.4 ± 0.5	

High-density lipoprotein (mg/dL)	-2.3 ± 7	-1.7 ± 6	0.37	-1.9 ± 7.0	-2.4 ± 6.0	0.50
Cholesterol (mmol/L)	-0.1 ± 0.2	-0.0 ± 0.2		-0.0 ± 0.2	-0.1 ± 0.2	

Low-density lipoprotein (mg/dL)	-17 ± 21	-10 ± 21	< 0.001^b^	-17 ± 22	-8 ± 19	< 0.001^b^
Cholesterol (mmol/L)	-0.5 ± 0.6	-0.3 ± 0.5		-0.4 ± 0.6	-0.2 ± 0.5	

^a ^Statistics represent N (%) and mean ± SD. *P *values from the chi-square test or t-test, as appropriate.

^b ^Significant if *P *< 0.05 (no correction testing at multiple weeks, to be conservative).

### Biometric changes

**Figure 1:**
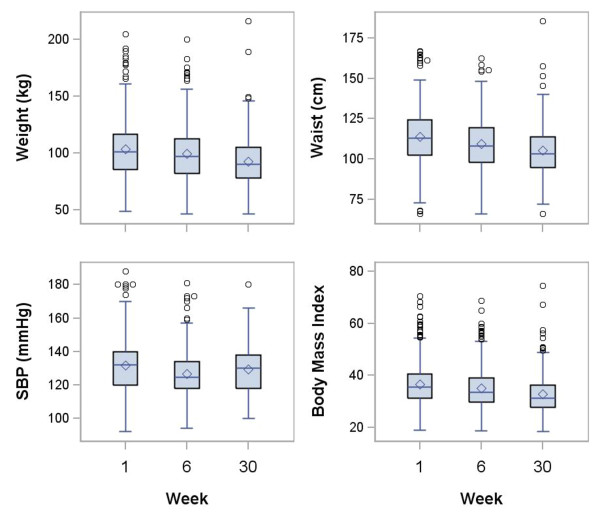
**Boxplots comparing weeks 1, 6 and 30 in each biometric variable using all non-missing data**. SBP denotes systolic blood pressure (mmHg). Box shows the interquartile range; horizontal line marks the median; whiskers extend to high and low values within 1.5 interquartile range of the box; circles are values beyond 1.5 interquartile ranges of the box; diamond shows the mean.

Table 3 presents descriptive statistics between baseline, 6 and 30 weeks (Figure 1). Mean weight change over 30 weeks was 6.8 ± 6.9 kg (Table 4). Of 244 participants who were measured at both baseline and 30 weeks, 183 (75%) had 2.3 kg or more weight loss, 66 (27%) had over a 5% weight loss and 67 additional (28%) had over a 10% weight loss; 24 (10%) of participants gained weight. The mean body mass index at 30 weeks was 33 ± 8 kg/m^2^, and mean waist circumference was 105 ± 16 cm. The mean ± SD systolic blood pressure at 30 weeks was 129 ± 14 mmHg. All biometric changes at 6 and 30 weeks were significantly decreased from baseline (paired t-test, all P < 0.001, except systolic blood pressure (P = 0.022)) (Table 4).

**Table 3:** Biometric outcomes: descriptive statistics by week of follow-up

Factor	Week One (N = 429)		Week Six (N = 404)		Week 30 (N = 244)	
	
	Mean ± SD	Min-Max	Mean ± SD	Min-Max	Mean ± SD	Min-Max
Weight (kg)	103 ± 25	49 - 205	99 ± 24	46 - 200	92 ± 22^a^	46 - 216
Waist (cm)	114 ± 18^a^	66 - 167	109 ± 16^b^	66 - 163	105 ± 16^c^	66 - 186
SBP (mmHg)	132 ± 15	92 - 188	127 ± 14	94 - 181	129 ± 14^b^	100 - 180
DBP(mmHg)	82 ± 10	50 - 120	79 ± 8	48 - 112	79 ± 8^b^	58 - 98
HR (bpm)	78 ± 11	52 - 114	72 ± 12^a^	43 - 115	71 ± 10	40 - 108
BMI (kg/m^2^)	37 ± 8	19 - 70	35 ± 8	19 - 69	33 ± 8^a^	18 - 74

Abbreviations: BMI, body mass index.

^a, b, c ^indicate 1, 2, and 6 missing data points, respectively.

**Table 4:** Biometric outcomes: changes from baseline to six and thirty weeks

Factor	Week 6 change from baseline (N = 404)		Week 30 change from baseline (N = 244)	
	
	Mean ± SD	*P *value^a^	Mean ± SD	*P *value^a^
Weight (kg)	-3.8 ± 2.6	< 0.001	-6.8 ± 6.9^c^	< 0.001
Waist (cm)	-4.2 ± 4.2^b^	< 0.001	-6.1 ± 7.3^d^	< 0.001
SBP (mmHg)	-5.1 ± 15.5	< 0.001	-2.5 ± 17.1^e^	0.022
DBP(mmHg)	-3.2 ± 9.6	< 0.001	-2.5 ± 10.0^e^	< 0.001
HR (bpm)	-5.3 ± 11.5^c^	< 0.001	-5.3 ± 11.5	< 0.001
BMI (kg/m^2^)	-1.3 ± 0.9	< 0.001	-2.4 ± 2.4^c^	< 0.001

Abbreviations: BMI, body mass index; SBP, systolic blood pressure; DBP, diastolic blood pressure; HR, heart rate.

**^a ^***P *value from paired t-test, significant if *P *< 0.025.

^b, c, d, e ^indicate 3, 1, 6 and 2 missing data points, respectively.

### Metabolic changes

**Figure 2:**
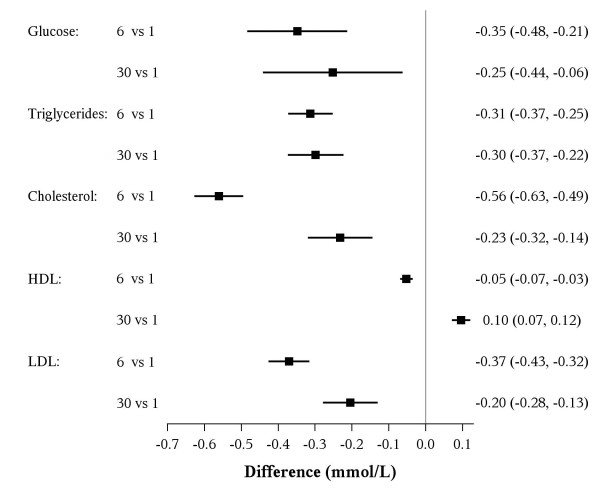
**Mean change (97.5% CI) in metabolic outcomes at six and thirty weeks from baseline**. Abbreviations: HDL, high-density lipoprotein cholesterol; LDL, low-density lipoprotein cholesterol.

Results for metabolic measures are presented in Table 5 at each point in time, while changes from baseline are shown in Table 6 and Figure 2. Means of all metabolic measures were significantly lower at 6 weeks compared with baseline (all P < 0.025). Mean glucose, total cholesterol, triglycerides, LDL, HgbA1c, insulin and US-CRP were also significantly lower at 30 weeks compared with baseline, while mean HDL levels rose 3.7 ± 8.4 mg/dL (0.10 ± 0.22 mmol/L) (P < 0.001). In general, changes at 30 weeks were sustained, but sometimes less than at 6 weeks.

**Table 5:** Metabolic outcomes by week^a^

Factor	Units	Week One (N = 423)		Week Six (N = 396)		Week 30 (N = 299)	
		
		Mean ± SD	Min - Max	Mean ± SD	Min - Max	Mean ± SD	Min -Max
Glucose	(mg/dL)	108 ± 35^b^	67 - 330	102 ± 27^c^	62 - 264	103 ± 29	59 - 339
	(mmol/L)	6.0 ± 1.9	3.7 - 18.3	5.7 ± 1.5	3.4 - 14.7	5.7 ± 1.6	3.3 - 18.8

Triglycerides	(mg/dL)	137 ± 81	34 - 839	107 ± 51	34 - 405	109 ± 52^d^	32 - 331
	(mmol/L)	1.5 ± 0.9	0.4 - 9.5	1.2 ± 0.6	0.4 - 4.6	1.2 ± 0.6	0.4 - 3.7

Cholesterol	(mg/dL)	187 ± 39	82 - 307	166 ± 36	80 - 294	179 ± 38^d^	93 - 290
	(mmol/L)	4.8 ± 1.0	2.1 - 7.9	4.3 ± 0.9	2.1 - 7.6	4.6 ± 1.0	2.4 - 7.5

HDL	(mg/dL)	51 ± 15	24 - 128	50 ± 14	21 - 110	55 ± 16^d^	21 - 147
	(mmol/L)	1.3 ± 0.4	0.6 - 3.3	1.3 ± 0.4	0.5 - 2.8	1.4 ± 0.4	0.5 - 3.8

LDL	(mg/dL)	109 ± 33^b^	21 - 218	95 ± 31	17 - 225	102 ± 32^e^	26 - 215
	(mmol/L)	2.8 ± 0.9	0.5 - 5.6	2.5 ± 0.8	0.4 - 5.8	2.6 ± 0.8	0.7 - 5.6

HgbA1c	(%)	6 ± 1^c^	5 - 13	N/A	N/A	6 ± 1^f^	5 - 10

Insulin	(microU/dL)	19 ± 18	3 - 134	14 ± 14	1 - 126	13 ± 10	0.5 - 72
	(pmol/L)	131 ± 122	17 - 930	97 ± 97	7 - 877	87 ± 73	3.5 - 500

US-CRP	(mg/mL)	5 ± 8	0.1 - 88	4 ± 5	0.2 - 33	4 ± 6	0.2 - 39
	(nmol/L)	45 ± 66	0.8 - 740	33 ± 45	1.7 - 278	33 ± 48	1.7 - 331

Abbreviations: HDL, high-density lipoprotein cholesterol; LDL, low-density lipoprotein cholesterol; HgbA1c, hemoglobin A1c; US-CRP, ultra sensitive c-reactive protein. N/A, no required measurement at six weeks.

^a ^N = 271, 266 and 185 for insulin and N = 285, 273 and 201 for US-CRP at weeks 1, 6, and 30, respectively, since neither parameter measured before June 2009;

^b, c, d, e, f ^indicate 3, 28, 7, 2, 1 and 15 missing data points, respectively.

**Table 6:** Metabolic outcomes: changes from baseline to six and thirty weeks^a^

F **Factor**	Units	Change at week six (N = 392)		Change at week thirty (N = 295)	
		
		Mean ± SD	**P value**^b^	Mean ± SD	**P value **^b^
Glucose	(mg/dL)	-6.3 ± 24.3^c^	< 0.001	-4.5 ± 29.6^e^	0.009
	(mmol/L)	-0.35 ± 1.35		-0.25 ± 1.64	

Triglycerides	(mg/dL)	-27.7 ± 53.8	< 0.001	-26.4 ± 58.5	< 0.001
	(mmol/L)	-0.31 ± 0.61		-0.30 ± 0.66	

Cholesterol	(mg/dL)	-21.7 ± 25.9	< 0.001	-9.0 ± 29.5	< 0.001
	(mmol/L)	-0.56 ± 0.67		-0.23 ± 0.76	

HDL	(mg/dL)	-2.0 ± 6.8	< 0.001	3.7 ± 8.4	< 0.001
	(mmol/L)	-0.05 ± 0.17		0.10 ± 0.22	

LDL	(mg/dL)	-14.3 ± 21.6^d^	< 0.001	-7.9 ± 25.1	< 0.001
	(mmol/L)	-0.37 ± 0.56		-0.20 ± 0.65	

HgbA1c	(%)	N/A	N/A	-0.2 ± 0.6^f^	< 0.001

Insulin	(microU/dL)	-4.4 ± 9.7	< 0.001	-3.8 ± 11.0	< 0.001
	(pmol/L)	-30.7 ± 67.4		-26.6 ± 76.4	

US-CRP	(mg/mL)	-1.4 ± 6.4	< 0.001	-0.9 ± 4.8	0.012
	(nmol/L)	-11.9 ± 54.0		-7.3 ± 40.2	

Abbreviations: HDL, high-density lipoprotein cholesterol; LDL, low-density lipoprotein cholesterol; HgbA1c, hemoglobin A1c; US-CRP, ultra sensitive c-reactive protein. N/A, no required measurement at six weeks.

^a ^N = 250, 173 for insulin and N = 270, 198 for US-CRP since neither measured before June 2009.

^b ^*P *value from the paired t-test, significant if *P *< 0.025.

^c, d, e, f ^indicate 9, 1, 3, and 24 missing data points, respectively.

### Change in metabolic syndrome status

By International Diabetes Federation criteria [14], 247 (59%) out of 422 participants were identified as having metabolic syndrome at baseline. Of the 382 participants for whom metabolic syndrome status (yes or no) could be determined at both baseline and 6 weeks, the percent with metabolic syndrome dropped from 58% (223/382) at baseline to 44% (167/382) at 6 weeks (McNemar's test, P < 0.001). Similarly, the percent with metabolic syndrome dropped from 54% (138/257) at baseline to 37% (94/257) at 30 weeks (P < 0.001).

### Relationship between clinical conditions and weight loss

Table 7 shows the relationship between clinical conditions and weight loss at 6 and 30 weeks from baseline. Mean weight loss was higher in the obese than in non-obese participants at both 6 weeks (4.2 ± 2.7 vs. 2.3 ± 1.9 kg, multivariable P < 0.001) and 30 weeks (7.6 ± 7.4 vs. 4.2 ± 4.1 kg, multivariable P < 0.001). However, there was no difference in mean percent weight loss between the obese and non-obese participants at either 6 weeks (multivariable P = 0.033) or 30 weeks (multivariable P = 0.038). The factors diabetic, hyperlipidemia, hypertension, and metabolic syndrome were not associated with the weight loss at 6 and 30 weeks (All P > 0.025).

**Table 7:** Relationship between baseline conditions and weight change (kg) at 6 and 30 weeks

Factor	Levels	N	Mean ± SD	Difference in **means (95% CI)**^**c**^	***P *value**^**c**^
**At 6 weeks^a^:**					

Diabetes	Yes	292	-3.9 ± 2.7	-0.11 (-0.72, 0.50)	0.73
	No	112	-3.7 ± 2.5		

Hyperlipidemia	Yes	243	-3.8 ± 2.6	-0.01 (-0.51, 0.54)	0.96
	No	161	-3.8 ± 2.8		

Hypertension	Yes	254	-3.9 ± 2.7	0.04 (-0.49, 0.57)	0.88
	No	150	-3.7 ± 2.6		

Metabolic syndrome	Yes	234	-4.3 ± 2.7	-0.57 (-1.13, -0.01)	0.048
	No	164	-3.2 ± 2.3		

Obesity (BMI > 30)	Yes	324	-4.2 ± 2.7	-1.61 (-2.26, -0.95)	< 0.001
	No	80	-2.3 ± 1.9	-0.64 (-1.24, -0.05) **^d^**	0.033

**At 30 weeks^b^:**					

Diabetes	Yes	176	-6.4 ± 6.6	1.22 (-0.91, 3.35)	0.26
	No	67	-7.7 ± 7.7		

Hyperlipidemia	Yes	151	-6.1 ± 6.7	1.51 (-0.36, 3.39)	0.11
	No	92	-7.9 ± 7.0		

Hypertension	Yes	155	-6.7 ± 7.1	0.32 (-1.60, 2.22)	0.74
	No	88	-6.9 ± 6.6		

Metabolic syndrome	Yes	135	-7.3 ± 8.1	-0.69 (-2.63, 1.25)	0.48
	No	104	-6.0 ± 4.9		

Obesity (BMI > 30)	Yes	185	-7.6 ± 7.4	-3.69 (-5.86, -1.52)	< 0.001
	No	58	-4.2 ± 4.1	-2.1 (-4.08, -0.12) **^d^**	0.038

**^a ^**Multivariable model (N = 398) including all five conditions in table plus age, gender, payment options.

**^b ^**Multivariable model (N = 239) including all five conditions in table plus age, gender, payment options.

**^c ^**Multivariable model results; significant if *P *< 0.025; difference in mean change for "Yes" minus "No".

**^d ^**Difference in mean percent change from baseline.

### Change in medication use

The summary of self-reported changes in medications for diabetes, hyperlipidemia and hypertension at week 30 is shown in Table 8. Participants' primary physicians adjusted medications (not the Lifestyle 180 medical team) as deemed necessary. Medication changes included beneficial categories of a) stopped medication (151 medications), b) decreased dose of medication (89 medications) or c) avoided medication (24 medications). Participants in the "avoided" category had, for example, abnormally elevated LDL cholesterol or hemoglobin A1c or were diagnosed with hypertension at baseline and were advised by their primary care physicians to start taking medications, but they refused. At week 30 their blood tests, or blood pressure were within normal ranges and they thus avoided the need to use a particular medication. On the opposite side, there were participants whose baseline or week 6 laboratory findings and blood pressure values were consistently abnormal or did not improve thus requiring that they start taking new medications (a total of 62 medications, 36 of those for uncontrolled hypertension) or increase the dose of their current medication(s) (18 medications). A total of 35 medication changes were made in the same category (for example, a diuretic was replaced with a calcium channel blocker) (data not shown). As shown in Table 8, for every newly started medication or one with increased dose, 3.3 medications were stopped, reduced in dose or avoided. This ratio was even better (4.0) in case of diabetes and hyperlipidemia medications.

**Table 8:** Medication changes at 30 weeks

	Diabetes	Hyperlipidemia	Hypertension	Total
Stopped	33	39	79	151
Decreased	47	12	30	89
Avoided	3	18	3	24
**Total**	**83**	**69**	**112**	**264**
Started	12	14	36	62
Increased	9	3	6	18
**Total**	**21**	**17**	**42**	**80**

## Discussion

Preventable chronic diseases continue to drive health care costs substantially and importantly upward, in part due to a lack of sustainable treatment options. Specific medications, such as cholesterol-lowering and diabetes medications target mostly one chronic disease-associated abnormality of elevated LDL cholesterol or plasma glucose, respectively, rather than the cause of the abnormality. While surgical interventions successfully treat obesity and a spectrum of associated metabolic changes, they poorly address the behavioral and lifestyle-related factors that led to the development of chronic conditions in the first place. In contrast, comprehensive lifestyle interventions may result in multiple physiological systems changes including behavior modifications that support long-term healthier lifestyle choices [5,29].

Here, we report findings that a tri-pronged lifestyle intervention consisting of diet, physical activity and stress management improves disease-associated markers of participants with multiple chronic conditions. All measured biometric and laboratory variables significantly improved after just 6 weeks of intervention. Beneficial changes in blood pressure and glucose were observed in many participants by the second week of the program, thus supporting previously reported observations of the quick onset of measurable benefits that follow a comprehensive lifestyle intervention [8]. Serum HDL cholesterol decrease after 6 weeks was most likely associated with early weight loss, as reported [30]. Nevertheless, reductions in total cholesterol are more profound than in HDL cholesterol, resulting in improved ratio of total cholesterol to HDL cholesterol, an established risk factor for coronary artery disease [31]. Although HDL cholesterol generally decreases during weight loss, it increases following weight maintenance in proportion to the amount of weight that is lost [32,33]. At week 30, HDL cholesterol is significantly higher than at the baseline and the total cholesterol/HDL ratio improves further. Fasting triglyceride level is also significantly reduced at both points in time (week 6 and 30) compared with baseline, reflecting the observations that triglycerides improve after weight loss [34]. Fasting hypertriglyceridemia is an established risk factor for cardiovascular disease and may predict disease progression [35]. In addition, TG/HDL ratio, a possible powerful predictor of extensive coronary artery disease is also improved, that is, decreased significantly [36]. In our study, we did not examine if these beneficial changes lead to better cardiovascular outcomes. In a comprehensive lifestyle intervention similar to ours, where a low-fat plant-based diet rather then a Mediterranean-style diet was used, reductions in total and LDL cholesterol were of greater magnitude than in this study and were associated with the arrest and/or reversal of coronary artery disease [5,37].

A large proportion of the global burden of chronic diseases, particularly cardiovascular disease, obesity and some cancers, involves non-resolving, chronic inflammation [38]. Lifestyle 180 intervention resulted in significantly healthier markers of glucose metabolism and inflammation (Table 6). Although measurements of insulin and US-CRP were initiated at later stages of the program and data were available for smaller number of participants, these results show large percentage decreases, strengthening the evidence that these lifestyle interventions may be used as anti-inflammatory therapies to treat insulin resistance, beneficially impact metabolic syndrome and associated chronic diseases [39]. It is, therefore, not surprising that the percent of participants with metabolic syndrome was significantly lower after 6 months of lifestyle interventions (an estimated relative 32 percent lower, from 54% to 37%).

Here we report only the outcomes for those participants who had biometric and lab measurements. These participants had greater lowering of mean weight and cholesterol at week 6 than those who did not attend this follow up. Thus, it is likely that those who witnessed greater early successes in their outcomes were more motivated to remain engaged in the program. We were unable to obtain biometric and laboratory measurements of patients who dropped out at 6 months, but the observed differences at 6 weeks suggest that those patients who dropped out at 6 months may have done so because their outcomes were not as good as for those who remained. At six months, 30-43% attrition rate (laboratory data and biometrics, respectively) was comparable to some lifestyle intervention programs, but lower than in others [40-42]. More than 75% of participants who enrolled into the program were selected by their employers on the basis that they had at least one of the chronic conditions and frequently included those who made little health improvements after participating in standard disease management programs. No pre-enrollment evaluation was conducted to ascertain whether potential participants were considering making lifestyle changes and whether they could count on support of their spouses and families. Local self-insured employers paid in full the costs of the program for their participants (with exception of 26 participants who paid 20% of the costs). Participants were not penalized if they missed the classes and no financial incentives (besides paying for the program itself) were provided to them to attend the classes. It is possible that the attendance at week 30 follow up would have been better if some of these factors were considered and follow up visits were more frequent, as evidenced by changes to the program (for example, shorter Immersion phase of 4 weeks instead of original 6 weeks has decreased spacing between the visits, the longest being now 2 months instead of 3 months) we made (our subsequent data not reported here). Adherence to lifestyle interventions seems to increase with multiple follow-up visits [43].

This study has several weaknesses. Pre-post type of study is not suitable to establish causality and assess how much of the observed changes are specifically due to the program. A randomized clinical trial would be needed for that purpose. However, implementation of a comprehensive lifestyle intervention in a randomized fashion is hampered by preferences of participants (those who prefer to engage in lifestyle improvement activities and those who don't) and may suffer from crossover problems [37]. Some of the observed changes could have been due to the placebo effect and possibly some other unidentified factors, not just to the participation in the lifestyle program per se.

Less than 25% of participants paid out of their pocket for participation in the program. This self-selected group of people who could financially afford the program and were able to accommodate their schedules during the first six weeks of the Immersion Phase are clearly not representative of the general population. For example, personality traits differ between obese persons who enroll and those who do not enroll in comprehensive lifestyle intervention programs [44]. Also, there was a bias in selection of participants by their employers to enroll in Lifestyle 180 as discussed above.

Because of only 6 months of intervention, inferences about long-term effectiveness cannot be made. Longer term data are needed to demonstrate that beneficial lifestyle changes are sustainable for such a large percentage of participants. An additional weakness of this study is that we did not evaluate adherence to the program and therefore we were unable to correlate the degree of adherence to the lifestyle program with changes in risk factors and to identify aspect(s) of lifestyle changes that are the most important for good outcomes. The outcomes data are reported for a heterogeneous group of participants and a subgroup analysis would be needed for better evaluation of chronic disease-specific outcomes. In addition, one might argue that significant improvements in lipids, glucose and inflammation markers could be the result of increased use of medications rather than participation in lifestyle intervention. While the cost-effectiveness analysis of the Lifestyle 180 program (including changes in pharmaceuticals) is in progress (manuscript in preparation), as shown in Table 8, for every newly started medication or one with increased dose, 3.3 medications were stopped, reduced in dose or avoided. This suggests that a spectrum of significant and beneficial biometric and biomarker improvements occurred among participants who used less, not more medications.

## Conclusions

Participation in a comprehensive Lifestyle 180 program results in significant and rapid, as well as clinically and biologically relevant improvements in biometric and laboratory outcomes, including reduced need for medications, for adults with multiple chronic conditions. Further follow up is needed to see if these beneficial changes are sustained.

## List of Abbreviations

LDL: low-density lipoprotein cholesterol; HDL: high-density lipoprotein cholesterol; HgbA1c: hemoglobin A1c; US - CRP: ultra sensitive C-reactive protein; SBP: systolic blood pressure.

## Competing interests

The authors have no potential conflicts of interest, including specific financial interests and relationships and affiliations relevant to the subject matter or materials discussed in the manuscript.

## Authors' contributions

All authors read and approved the final manuscript. Study concept and design: EWR, MFR, LS; Acquisition of data: EWR, MG; Statistical analysis and interpretation of data: EM, DY, MFR, MG; Drafting of the manuscript: EWR, MG, EM, MFR, DY; Critical revision of the manuscript for important intellectual content: MFR, LS, MG; Administrative, technical, or material support: MFR, EWR..
